# Diastolic Function in Paced Children with Cardiac Defects: Septum vs Apex

**DOI:** 10.5935/abc.20150077

**Published:** 2015-08

**Authors:** Michel Cabrera Ortega, Adel Eladio Gonzalez Morejon, Giselle Ricardo Serrano, Dunia Barbara Benitez Ramos

**Affiliations:** Cardiocentro Pediatrico William Soler, La Habana - Cuba

**Keywords:** Heart Defects, Congenital, Ventricular Function, Right, Ventricular Function, Left, Child, Pacemaker, Artificial

## Abstract

In children with structural congenital heart disease (CHD), the effects of chronic ventricular pacing on diastolic function are not well known. On the other hand, the beneficial effect of septal pacing over apical pacing is still controversial.

The aim of this study was to evaluate the influence of different right ventricular (RV) pacing site on left ventricular (LV) diastolic function in children with cardiac defects.

Twenty-nine pediatric patients with complete atrioventricular block (CAVB) and CHD undergoing permanent pacing were prospectively studied. Pacing sites were RV apex (n = 16) and RV septum (n = 13). Echocardiographic assessment was performed before pacemaker implantation and after it, during a mean follow‑up of 4.9 years.

Compared to RV septum, transmitral E-wave was significantly affected in RV apical pacing (95.38 ± 9.19 vs 83 ± 18.75, p = 0.038). Likewise, parameters at the lateral annular tissue Doppler imaging (TDI) were significantly affected in children paced at the RV apex. The E´ wave correlated inversely with TDI lateral myocardial performance index (Tei index) (R^2^= 0.9849, p ≤ 0.001). RV apex pacing (*Odds ratio*, 0.648; confidence interval, 0.067-0.652; p = 0.003) and TDI lateral Tei index (*Odds ratio*, 31.21; confidence interval, 54.6-177.4; p = 0.025) predicted significantly decreased LV diastolic function.

Of the two sites studied, RV septum prevents pacing-induced reduction of LV diastolic function.

## Introduction

RV apical pacing is conventionally performed in pediatric patients with CAVB. However, ventricular pacing induces an abnormal electrical activation pattern, which causes mechanical dyssynchrony, LV structural remodeling and increased risk of heart failure^1-3^. Most pediatric studies published^1,2^have focused on ventricular systolic function assessment; therefore, the effects of chronic ventricular pacing on diastolic function are not well known, even less in children with CHD.

Moreover, the benefit of RV septal stimulation is still controversial, with clinical studies^4^ showing promising results, while a recent research did not demonstrate any superiority over RV apical pacing in children^1^; none of these studies^1,2,4^reported the effects on LV relaxation phase.

With the hypothetical premise that there are differences between RV septal and RV apical pacing in terms of dynamic alterations in LV filling, we performed the current study.

## Methods

The study included all children with CHD and CAVB that underwent pacemaker implantation in a single tertiary pediatric cardiology center, paced from RV septum (n = 13) and from RV apex (n = 16). Patients with clinical or anamnestic evidence of heart failure were excluded. None of the patients were older than 18 years at pacemaker implantation, had ≤ 95% of ventricular pacing or ≤ 1 year of permanent cardiac pacing. The study protocol was approved by the institutional research ethics committee and parental written consent was obtained.

Two experienced observers, blinded for the ventricular pacing site, performed prospective echocardiographic evaluations (Aloka α-10) before pacemaker implantation, immediately after and regularly during a mean period of 4.9 years. Three random measurements were made for every patient by each observer and the average of measurements was used for further analysis. For a comprehensive diastolic evaluation, the following mitral flow parameters were evaluated by pulsed wave Doppler echocardiography: E and A waves, E/A wave ratio and E-wave deceleration time. Likewise, pulse wave TDI velocities were obtained in the apical four-chamber view, at septal and lateral mitral annulus. In each segment, peak systolic (S´), early (E´) and late (A´) peak diastolic velocities were measured. The E/E´ ratio and TDI Tei index were also calculated. All data were prospectively collected.

### Statistical analysis

According to the Kolmogorov-Smirnov test, the variables that showed a normal distribution were summarized as mean ± standard deviation. The differences between two groups were compared by unpaired *t*-test. Independent variables showing significant univariate differences related to the development of LV dysfunction were entered into a backward stepwise logistic regression analysis, where the *Odds ratio *(OR) and Wald statistics for each variable were identified. Significance level was set at 5%. The statistical software Medcalc Version 12 was used for the analyses.

## Results

A total of 29 patients (surgical atrioventricular block in 26), with mean age at first implantation of 9.82 ± 2.75 years were evaluated. Tetralogy of Fallot (8 cases, 27%) and ventricular septal defect (7 patients, 24.13%) were the main CHD corrected before pacemaker implantation. Anatomic surgical correction was performed in all patients and mild residual atrioventricular regurgitation was present in 10 (34.48%) children. Thirteen (44.82%) cases underwent treatment with angiotensin‑converting enzyme inhibitors at the time of implantation. Twelve children (41.37%) received a single‑chamber pacemaker, while 11 (24.13%) patients underwent DDD/DDDR pacing. Mean pacing duration was 4.9 years.

**Figure 1:**
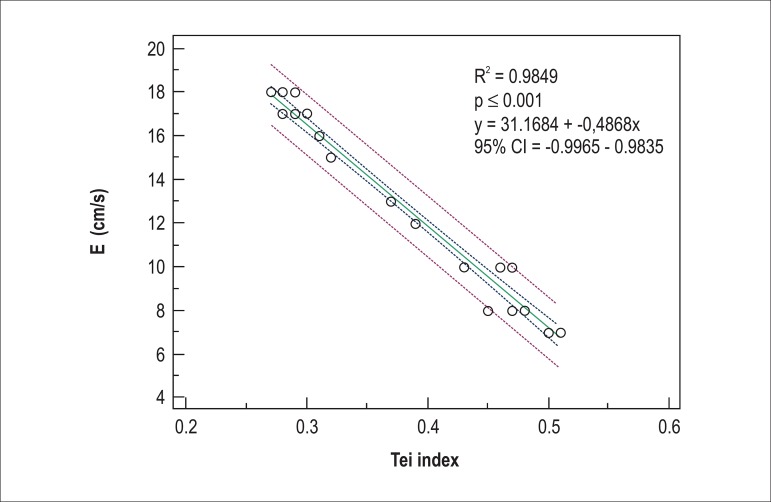
Association between E´-wave and TDI Tei index at lateral mitral valve annulus.

Compared to RV septum, transmitral E-wave was significantly affected in RV apical pacing (95.38 ± 9.19 vs 83 ± 18.75, p = 0.038) (Table 1). Likewise, the following parameters of the lateral annular TDI were significantly affected in children paced at the RV apex compared with RV septum group: E´ wave (12.5 ± 4.42 vs 15.3 ± 2.1; p = 0.046), A´ wave (8.12 ± 2.63 vs 6.22 ± 2.11; p = 0.045), E/E´ ratio (8.2 ± 1.29 vs 6.3 ± 0.72; p = 0.0001) and Tei index (0.39 ± 0.04 vs 0.34 ± 0.04; p = 0.002). The E´ wave correlated inversely with TDI lateral Tei index (R^2^ = 0.9849, p ≤ 0.001) (Figure 1). At the logistic regression, pacing from the RV apex (*OR*, 0.648; confidence interval, 0.067-0.652; Wald, -0.915; p = 0.003) and TDI lateral Tei index (*OR*, 31.21; confidence interval, 54.6-177.4; Wald, 3.046; p = 0.025) predicted significantly decreased LV diastolic function.

**Table 1:** Comparison of LV function between RV septal and apical pacing

	**RV Septum (n = 13)**			**RV Apex (n = 16)**			p*
	**Before PM implantation**	**At last follow-up**	**p**	**Before PM implantation**	**At lastfollow-up**	**p**	
**LVEF**	64.16 ± 1.75	61.43 ± 2.26	0.004	65.21 ± 2.08	64.22 ± 3.14	0.352	0.009
**Mitral Inflow Doppler indices**							
E(cm/s)	90.72 ± 13.81	95.38 ± 9.19	0.321	90.53 ± 11.45	83 ± 18.75	0.181	0.038
A(cm/s)	61.46 ± 15.19	56.69 ± 7.2	0.316	65.87 ± 18.31	67.06 ± 19.66	0.860	0.100
E/A	1.59 ± 0.51	1.71 ± 0.33	0.483	1.51 ± 0.54	1.41 ± 0.65	0.639	0.142
EDT(ms)	170.38 ± 23.54	172.07 ± 17.45	0.837	170.68 ± 24.06	172.8 ± 25.84	0.811	0.931
**Lateral Mitral Valve Annular TDI**							
E´(cm/s)	15 ± 3.41	15.3 ± 2.1	0.789	15.6 ± 3.31	12.5 ± 4.42	0.032	0.046
A´(cm/s)	6.41 ± 2.13	6.22 ± 2.1	0.820	7.1 ± 2.11	8.12 ± 2.63	0.235	0.045
E/E´	6.1 ± 0.81	6.3 ± 0.72	0.512	5.8 ± 0.62	8.2 ± 1.29	< 0.0001	0.0001
Tei index	0.33 ± 0.04	0.34 ± 0.04	0.529	0.35 ± 0.05	0.39 ± 0.04	0.018	0.002
**Septal Mitral Valve Annular TDI**							
E´(cm/s)	15.30 ± 4.23	14.84 ± 3.51	0.765	15.12 ± 3.28	13.81 ± 3.97	0.317	0.470
A´(cm/s)	7.23 ± 2.35	7.24 ± 2.33	0.991	7 ± 2.55	6.56 ± 2.65	0.616	0.474
E/E´	6.19 ± 1.11	6.68 ± 1.22	0.294	6.11 ± 0.74	6.13 ± 0.56	0.931	0.118
Tei index	0.34 ± 0.06	0.35 ± 0.04	0.621	0.33 ± 0.01	0.36 ± 0.08	0.147	0.685

Data expressed by mean ± standard error.

p*: septum vs. apex at last follow-up.

EDT: E-wave deceleration time; LVEF: Left ventricular ejection fraction; PM: Pacemaker; RV: Right ventricular; TDI: Tissue doppler imaging.

## Discussion

Our study confirms that chronic stimulation from RV apex results in diastolic function impairment in pediatric patients with CHD and further demonstrates the superiority of septal stimulation in this context.

The deterioration of diastolic function after RV pacing has been previously reported in animals^5^ and in the adult population^6,7^. Aoyagi et al^5^ showed that wall motion asynchrony prolongs LV isovolumic relaxation time (IVRT) in dogs; this impairment correlated with the degree of wall motion asynchrony. In the research performed by Kolettis et al^6^, compared to RV outflow tract pacing, RV apical pacing decreased maximum negative dp/dt and increased the IVRT. These findings were confirmed in an analysis of nine studies^7^, reporting a significant benefit of RV outflow tract over apical pacing. On the other hand, few investigations^3,8,9^have focused on LV diastolic function in the pediatric population. Forwalt et al^8^ evaluated the effects of acute ventricular pacing in children who underwent ablation therapy; the authors observed that RV apical pacing resulted in acute systolic dyssynchrony with preserved diastolic synchrony. Nevertheless, Koh et al^9^ provided evidence of LV diastolic dysfunction after chronic RV apical stimulation, associated with the presence of LV dyssynchrony. In our study, the impaired diastolic indices in the lateral mitral annulus could be associated with the pattern induced by RV apical pacing, characterized by early activation of the RV and delayed activation of the LV lateral wall.

The Tei index has been used to assess LV function in a wide variety of diagnoses in children^10^; it is the most accurate for the detection of diastolic and combined dysfunction^10^. Considering that the results of our research reflect the high predictive value of this parameter, it could be used as an echocardiographic tool to predict the deterioration of both systolic and diastolic functions in patients with chronic ventricular pacing.

## Conclusions

Of the two assessed sites, RV septum showed to prevent pacing-induced reduction of LV diastolic function.
